# Improvement in CT image resolution due to the use of focal spot deflection and increased sampling

**DOI:** 10.1120/jacmp.v17i3.6039

**Published:** 2016-05-08

**Authors:** Nicholas Rubert, Timothy Szczykutowicz, Frank Ranallo

**Affiliations:** ^1^ Department of Medical Physics University of Wisconsin‐Madison Madison WI USA; ^2^ Department of Radiology University of Wisconsin‐Madison Madison WI USA; ^3^ Department of Biomedical Engineering University of Wisconsin‐Madison Madison WI USA

**Keywords:** MTF, focal spot deflection, resolution, high definition

## Abstract

When patient anatomy is positioned away from a CT scanner's isocenter, scans of limited diagnostic value may result. Yet in some cases, positioning of patient anatomy far from isocenter is unavoidable. This study examines the effect of position and reconstruction algorithm on image resolution achieved by a CT scanner operating in a high resolution (HR) scan mode which incorporates focal spot deflection and acquires an increased number of projections per rotation. Images of a metal bead contained in a phantom were acquired on a GE CT750 HD scanner with multiple reconstruction algorithms, in the normal and HR scan mode, and at two positions, scanner isocenter and 15 cm directly above isocenter. The images of the metal bead yielded two‐dimensional point spread functions which were averaged along two perpendicular directions to yield line spread functions. Fourier transforms of the line spread functions yielded radial and azimuthal modulation transfer functions (MTFs). At isocenter, the radial and azimuthal MTFs were averaged. MTF improvement depended on image position and modulation direction. The results from a single algorithm, Edge, can be generalized to other algorithms. At isocenter, the 10% MTF cutoff was 14.4 cycles/cm in normal and HR mode. At 15 cm above isocenter, the 10% cutoff was 6.0 and 8.5 cycles/cm for the azimuthal and radial MTFs in normal mode. In HR mode, the azimuthal and radial MTF 10% cutoff was 8.3 and 10.3 cycles/cm. Our results indicate that the best image resolution is achieved at scanner isocenter and that the azimuthal resolution degrades more significantly than the radial resolution. For the GE CT750 HD CT scanner, the resolution is significantly enhanced by the HR scan mode away from scanner isocenter, and the use of the HR scan mode has much more of an impact on image resolution away from isocenter than the choice of algorithm.

PACS number(s): 87.57.Q‐

## I. INTRODUCTION

Many applications in CT imaging demand excellent high‐contrast spatial resolution. For example, the ability to visualize small structures is necessary when trying to locate small pulmonary nodules,^(1)^ visualizing the temporal bone, imaging small caliber coronary artery stents,^2^, ^3^ or assessing bone fractures to determine a course of treatment.^(4)^ The resolution of a CT scanner may be divided into in‐plane spatial resolution and longitudinal spatial resolution. Authors frequently characterize the in‐plane spatial resolution in CT by presenting estimates of the modulation transfer function (MTF) for a CT scanner.^5^, ^6^, ^7^, ^8^, ^9^ When the reconstruction algorithm is filtered back‐projection, it is assumed that the two‐dimensional MTF describing in‐plane spatial resolution is independent of contrast level and dose, radially symmetric, and shift‐invariant. Therefore, when reconstructing images with filtered back‐projection, the MTF is presented as a single, 1D curve.^5^, ^6^, ^7^, ^8^, ^9^, ^10^

When examining iterative nonlinear reconstruction algorithms, authors have demonstrated that resolution is dependent on both contrast and dose level, and that different analysis techniques are necessary for describing resolution. For example, presenting multiple, task‐specific MTFs estimated at different contrast and dose levels has been considered.^(11)^ Additionally, characterizing resolution at different dose levels and contrast in terms of the point spread function rather than the MTF has been proposed.^(12)^ However, even considering filtered back‐projection only, a single MTF curve does not fully characterize the in‐plane high‐contrast spatial resolution of a scanner as the in‐plane resolution of a CT scanner is spatially variant and anisotropic.^(13)^ While it is common to present MTF measurements obtained at a single location only,^5^, ^6^, ^7^, ^8^, ^9^, ^10^, ^11^ an MTF measured by imaging an object located at or near isocenter will not reflect the resolution of a CT scanner in a region away from the scanner isocenter.^(13)^

**Figure 1:**
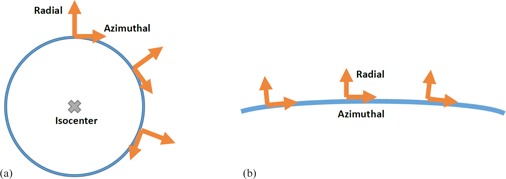
(a) Depiction of polar coordinate system. Radial basis vectors point radial outwards from a circle with center at scanner isocenter. Azimuthal vectors lay tangent to circle and perpendicular to radially directed vectors. (b) When examining a small arc length along a circle at a large distance from isocenter there is little change in direction of the radial and azimuthal coordinate vectors along that arc length.

While a point‐like object imaged at the isocenter of a CT scanner will blur in a radially symmetric manner, loss of image resolution in CT away from isocenter depends on different components of the underlying scanner hardware, depending on the direction of the blurring.^(13)^ Consider the radial versus the azimuthal direction within an imaging plane, as illustrated in Fig. 1(a). The radial direction is perpendicular to a circle with center at isocenter and the azimuthal direction lies tangent to this circle. In the radial direction, the resolution is affected by the projected sizes of the focal spot at the detector and the detector aperture at the X‐ray source.^(13)^ Meanwhile, the azimuthal resolution is affected by the number of projection views. The angle swept out during a single ray measurement increases with fewer projection views and, for most CT scanners, the beam is on continuously. Therefore, data are integrated over an angle determined by the number of projections. Azimuthal resolution varies with radial distance from isocenter because the arc length swept out by a single point on a ray during a sampling interval increases with distance from isocenter.^(13)^ This leads to the best resolution occurring at scanner isocenter, and the resolution suffering more in the azimuthal than in the radial direction at positions away from isocenter.

Focal spot deflection is one technique for increasing the number of projection views acquired. When focal spot deflection is used, back and forth movement of the X‐ray focal spot between two positions is timed so that rays are effectively measured at detector positions midway between the actual detector positions. In one particular implementation of focal spot deflection, two consecutive projection views are combined to create a single virtual projection view with a common focal spot and double sampling along the detector row.^(14)^ Focal spot deflection has two effects on image acquisition. First, the number of acquisitions per rotation is increased, the acquisition time for a single sample is decreased, and therefore azimuthal blurring is decreased. Additionally, the increased number of samples substantially suppresses aliasing artifacts.^(14)^

In‐plane focal spot deflection is not new technology^(15)^ and many manufacturers currently provide X‐ray tubes capable of focal spot deflection. One example of an X‐ray tube with focal spot deflection capabilities is Siemens' Somatom Force (Siemens Healthcare, Forchheim, Germany) with the Vectron X‐ray tube. This X‐ray tube allows for focal spot deflection both in‐plane and longitudinally. Both Philips' MRC800 X‐ray tube on the Brilliance CT scanners (Philips Medical Systems, Amsterdam, The Netherlands) and GE's Performix X‐ray tube on the Revolution HD and CT750 HD scanners (GE Healthcare, Waukesha, WI) allow for double sampling to achieve increased in‐plane spatial resolution.

This paper describes the improvement in image resolution for the CT750 HD scanner that is achieved by an imaging mode incorporating focal spot deflection compared to the normal imaging mode with a lower sampling rate and without focal spot deflection. There are several variables to consider when utilizing focal spot deflection to increase image resolution. The relative benefit to image resolution due to focal spot deflection and an increased number of projections depends on position within the imaging plane, choice of reconstruction options, and the direction of image blurring being considered. The relationship between these variables was examined by measuring MTF at scanner isocenter and at a position 15 cm away from scanner isocenter in both imaging modes, and utilizing a number of reconstruction options in each scan mode.

## II. MATERIALS AND METHODS

### A. CT Lexicon

This section provides a discussion of terminology used by GE scanner architecture. Henceforth, the scan mode which does not utilize focal spot deflection and acquires images at a lower resolution is referred to as the normal mode. The mode utilizing focal spot deflection which acquires higher resolution images is referred to as “High Resolution” (HR) mode. On a GE scanner, the parameter which describes the filter used for FBP reconstruction and therefore the trade‐off between resolution and noise is referred to as an Algorithm. This term is also clarified in the AAPM CT Lexicon.^(16)^ The AAPM CT Lexicon is a document from the AAPM Working Group on Standardization of CT Nomenclature and Protocols. Its purpose is to translate vendor‐specific terminology from one manufacturer to another. According to this document, the term Algorithm on a GE scanner refers to the “reconstruction property that determines sharpness or smoothness of image in the axial plane.” On the CT 750 HD scanner in the normal mode, the following Algorithms are available in approximate order of increasing resolution: Soft, Standard, Detail, Lung, Bone, Bone Plus, and Edge. In the HR mode another set of “High Definition” (HD) algorithms are available: HD Standard, HD Detail, HD Lung, HD Bone, HD Bone Plus, HD Ultra, and HD Edge.

### B. MTF: image acquisition

In this study, all images were acquired on a GE CT 750 HD scanner (GE Healthcare, Waukesha, WI). To estimate the MTF of the CT750 HD scanner, a tungsten carbide bead with a diameter of 0.28 mm contained in the resolution module of a Catphan 600 phantom (Phantom Laboratory, Salem, NY) was imaged. The bead was imaged in the axial scan mode with an image matrix of 512×512 pixels, a display field of view (DFOV) of 5.0 cm, a Medium Body scan field of view, a slice thickness of 5.0 mm, a beam width of 20 mm (detector configuration of 32×0.625), a rotation time of 2.0 s, a tube current of 300 mA, and a tube potential of 120 kV. Each scan was repeated 64 times, and the bead was scanned using both the HR mode and the normal mode. The ${\text{CTDI}}_{\text{vol}}$ was the same for HR and normal mode and was 27.41 mGy for each scan.

**Figure 2:**
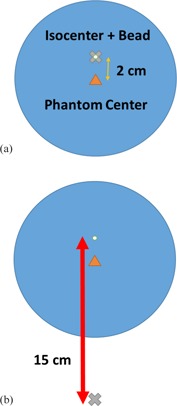
(a) Diagram of Catphan bead placed at isocenter; the phantom center is 2 cm below the bead. (b) Catphan with bead placed 15 cm directly anterior to isocenter. (Diagrams not drawn to scale.)

Images were acquired with the bead at two positions: isocenter and 15 cm above scanner isocenter. The bead was located 2 cm above the center of the phantom, so to position the bead at isocenter the center of the phantom was 2 cm below isocenter. The setup for the experiments is shown in Fig. 2(a) and (b). Images were reconstructed with the following Algorithms: Standard, Bone, Bone Plus, Edge, HD Bone, HD Bone Plus, and HD Edge. When scanning using the normal scan mode only non‐HD Algorithms can be used. When scanning using the HR scan mode, all Algorithms were used, both HD and non‐HD

Each of the 64 scans yielded a single image of the bead at a longitudinal position of z=0, giving 64 images of the bead for a given scan mode, bead position, and algorithm. Sets of six of these images were averaged to yield 10 images with lower noise for each experimental condition. Additionally, all 64 images were averaged for each experimental condition to yield a single image with low noise. An experimental condition refers to a particular scan mode (normal vs. HD), bead position, and Algorithm. The single low‐noise image using all 64 scans for a single experimental condition was used to calculate the MTF curves shown in 3, 5. The set of ten average images, each average image calculated using sets of six scans, was used for computing MTF cutoff values, as described in the next section, and displayed in 1, 3.

**Figure 3:**
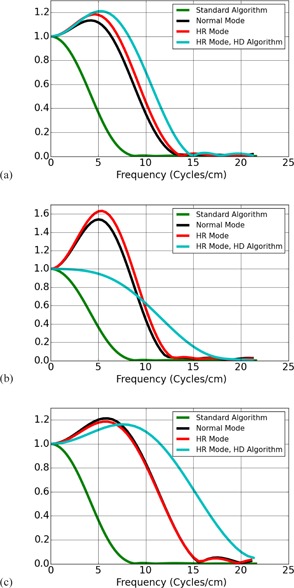
MTF measured at scanner isocenter with non‐HD algorithm applied in the normal resolution scan mode and the HR scan mode, and an HD reconstruction algorithm applied in the HR scan mode. The MTF estimated for the standard algorithm in the normal scan mode is included in all plots. MTF shown for (a) Bone and HD Bone, (b) Bone Plus and HD Bone Plus, and (c), Edge and HD Edge algorithms.

**Figure 4:**
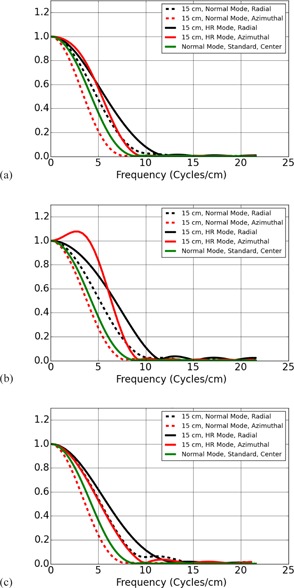
Normal resolution scan mode versus HR scan mode at 15 cm off isocenter for (a) Bone, (b) Bone Plus, and (c) Edge algorithms. Standard algorithm at isocenter included in each plot for comparison. Note that the bone, edge, and bone plus algorithms at 15 cm off isocenter perform comparably to the standard algorithm at isocenter. The HR scan modes shown in this figure did not use the HD algorithms.

**Figure 5:**
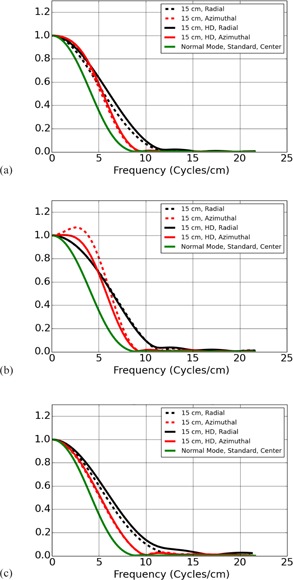
MTF plots at 15 cm away from isocenter in high‐resolution scan mode: (a) comparing Bone and HD Bone algorithms, (b) comparing Bone Plus and HD Bone Plus algorithms, (c) comparing Edge and HD Edge algorithms. MTF measurement of Standard Algorithm is at scanner isocenter.

**Table 1:** 10% MTF cutoff values measured at isocenter (cycles/cm) and radial and azimuthal 10% MTF cutoff values estimated at 15 cm off isocenter (cycles/cm) for Bone and HD Bone Algorithms. Values given as the sample mean ± the sample standard deviation.

	*Bone‐Centered*	*Bone‐Azimuthal MTF 15 cm off‐center*	*Bone‐Radial MTF 15 cm off‐center*
Normal Scan Mode	11.81±0.07	5.80±0.04	8.10±0.10
HR Scan Mode	12.28±0.04	7.88±0.05	9.70±0.36
HR Scan Mode+HD Algorithm	13.59±0.07	7.96±0.07	10.30±0.78

**Table 2:** 10% MTF cutoff values measured at isocenter (cycles/cm) and radial and azimuthal 10% MTF cutoff values estimated at 15 cm off isocenter (cycles/cm) for Bone Plus and HD Bone Plus Algorithms. Values given as the sample mean ± the standard error.

	*Bone Plus‐Centered*	*Bone Plus‐Azimuthal MTF 15 cm off‐center*	*Bone Plus‐Radial MTF 15 cm off‐center*
Normal Scan Mode	11.38±0.19	6.17±0.08	8.52±0.22
HR Scan Mode	12.10±0.07	8.18±0.08	10.18±0.47
HR Scan Mode+HD Algorithm	15.80±0.25	8.00±0.14	10.56±1.41

**Table 3:** 10% MTF cutoff values measured at isocenter (cycles/cm) and radial and azimuthal 10% MTF cutoff values estimated at 15 cm off isocenter (cycles/cm) for Edge and HD Edge Algorithms. Values given as the sample mean ± the sample standard deviation.

	*Edge‐Centered*	*Edge‐Azimuthal MTF 15 cm off‐center*	*Edge‐Radial MTF 15 cm off‐center*
Normal Scan Mode	14.43±0.07	6.03±0.14	8.51±0.43
HR Scan Mode	14.42±0.09	8.34±0.17	10.26±0.51
HR Scan Mode+HD Algorithm	19.89±0.45	7.84±1.12	12.01±1.93

### C. MTF calculations

The geometry of the MTF calculations is depicted in Fig. 1(b). If an imaginary circle is drawn away from isocenter, then the azimuthal direction lays tangent to this circle and the radial direction points perpendicularly out and away from this circle. We adopt the convention that the radial MTF represents modulation in the radial direction and the azimuthal MTF represents modulation in the azimuthal direction. These directions are with respect to the scanner isocenter. However, as illustrated in Fig. 1(b), when the bead is directly above isocenter the radial direction corresponds to the y‐axis of a local Cartesian coordinate system and the azimuthal direction corresponds to the x‐axis. For the calculations presented, the ROIs examined were less than 4 mm wide in either dimension and were at a distance of 15 cm from isocenter so the calculation ROI spanned an extent of approximately 2°. Therefore we associate the local y‐axis of the ROIs around the bead with the radial direction, and the local x‐axis of the ROI around the bead with the azimuthal direction.

To compute the radial or azimuthal MTF, each 512 by 512 low‐noise image was cropped to a 4 mm by 4 mm or smaller region centered on the tungsten carbide bead. The cropped, low noise image was further averaged across image rows (radial MTF) or image columns (azimuthal MTF) to yield a line spread function (LSF). A background value was determined from the mean pixel value in a 1 mm by 1 mm rectangle bordering the cropped region of the low‐noise image. The background value was subtracted from the LSF. After subtracting the background value, the LSF was normalized so that the MTF would have a value of 1 at f=0. Normalization was achieved by adding the value of the discrete LSF at each point and then dividing the LSF by this sum. The LSF was then zero‐padded by a factor of 10 in order to upsample the MTF. The absolute value of the discrete Fourier transform of the signal then yielded an MTF in either the azimuthal or the radial direction. All MTFs were divided by a sinc function to deconvolve the 0.28 mm bead. Specifically, the MTF was divided by the following function:

(1) Sinc(ak)=sin(πak)/(πak)

where *a* is the bead diameter of 0.28 mm and *k* is spatial frequency in cycles/mm.

**Figure 6:**
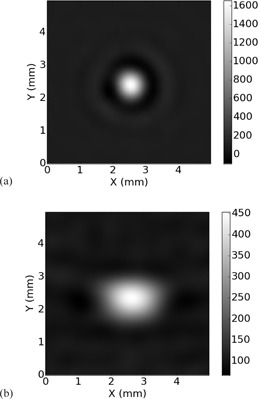
Image of the tungsten carbide bead, averaged over 64 scans. The Edge algorithm was used. (a) Bead positioned at isocenter. (b) Bead positioned 15 cm anterior to isocenter. Notice the bead has a symmetric, circular appearance at isocenter and is blurred more in the azimuthal than in the radial direction away from isocenter. Colorbar scale is in HU.

At isocenter, the radial and azimuthal MTF were assumed to be identical and were averaged to yield a single MTF. Figure 6 demonstrates the validity of this assumption. In Fig. 6(a) the bead exhibits a symmetric, circular appearance. In Fig. 6(b), the bead takes on a broader, elliptical appearance. The elliptical shape of the bead is due to the difference in the radial and azimuthal resolution. In addition to the change in the shape of the bead depicted in the image, there is also a large change in the peak value of the CT number due to image blurring away from isocenter. This is because the CT number of the background material is much lower than the CT number of the bead so that the blurring of the bead results in a lower peak CT number in the image.

Ten percent MTF cutoff values were determined for each experimental condition. An MTF cutoff value is the spatial frequency where the MTF fell to 0.1. For the MTF cutoff values, six images were used to compute an average image prior to computing MTF curves. The MTF cutoff was found by linearly interpolating between the two adjacent, discrete points where the MTF was above and below 0.1. This calculation was repeated 10 times in order to yield standard errors. Statistical significance for differences between select MTF cutoff values was computed by a standard two‐tailed *t*‐test for the difference of the mean of two groups of measurements. A statistically significant difference was considered to be 1, with a p‐value of less than .05

### D. Noise measurements

In addition to MTF measurements, noise measurements were made by imaging a uniform region of the Catphan 600 with phantom center at isocenter. The noise measurements were obtained in a uniform slice of the Catphan using a 40 mm^2^ circular ROI centered at the scanner isocenter. They were made with the same scan settings as the MTF measurements: a beam width of 20 mm (detector configuration of 32×0.625), a rotation time of 2.0 s, a tube current of 300 mA, a slice thickness of 5.0 mm, and a tube potential of 120 kV. However, the DFOV was changed to 12.0 cm to more accurately reflect the noise in a clinically useful DFOV. A pair of scans was acquired for each scan mode and Algorithm used in the MTF measurements. Image subtraction was performed for each pair, to eliminate any effect of image cupping or artifacts. All noise values reported in the results were calculated as the standard deviation (SD) of the ROI in the subtraction image divided by the square root of two to obtain the noise for a single image.

### E. Cadaveric human femur image acquisition

CT images of a cadaveric human femur were also acquired. All cadaver images were acquired in an axial scan mode with a collimation of 20 mm (detector configuration of 32×0.625), Medium Body SFOV, 100 kV, 500 mA, and a 2.0 s rotation time. This scan had a ${\text{CTDI}}_{\text{vol}}$ of 28.80 mGy. Two sets of cadaver images were acquired: one set using the normal scan mode and the other set in the HR scan mode. We compare select images from each acquisition in the discussion section.

## III. RESULTS

Figure 3 shows the MTF estimated for the different Algorithms and scan modes at scanner isocenter. There are three plots, each showing the normal scan mode with the (a) Bone, (b) Bone Plus, and (c) Edge Algorithms in separate plots, and the HR scan mode with the (a) Bone and HD Bone Plus, (b) Bone Plus and HD Bone Plus, and (c) Edge and HD Edge Algorithms. In each plot of MTFs displayed in this section, the MTF for the Standard Algorithm in the normal scan mode measured at isocenter is included for the sake of comparison. At scanner isocenter the plots in Fig. 3 show little difference between the HR scan mode and the normal scan mode for the Algorithms available to both modes. From 1, 3, when changing from normal scan mode to HR scan mode, the 10% MTF cutoff increased by approximately 3.98% and 6.33% for the Bone and Bone Plus Algorithm and remained unchanged for the Edge Algorithm. Meanwhile, the HD Algorithms provide a considerable increase in image resolution at isocenter, especially for the HD Edge kernel. From 1, 3, when comparing an Algorithm with its HD counterpart in HR scan mode, the 10% MTF cutoffs increase by approximately 10.67% for the HD Bone Algorithm, 30.58% for the HD Bone Plus Algorithm, and 37.93% for the HD Edge Algorithm.

Figure 4 displays the same set of MTFs measured 15 cm away from scanner isocenter. In all cases, the radial MTF is significantly better than the azimuthal MTF. From 1, 3, in the normal scan mode the 10% MTF cutoff for the Bone, Bone Plus, and Edge Algorithms is approximately 5.8, 6.2, and 6.0 cycles/cm in the azimuthal direction and 8.1, 8.5, and 8.5 cycles/cm in the radial direction. Examining Fig. 4, it can be seen that in the normal scan mode the azimuthal MTF lies below the MTF for the standard Algorithm at scanner isocenter for all three Algorithms. This result quantifies the asymmetric blurring demonstrated by the images of the bead shown in Fig. 6.

Away from isocenter, the HR scan mode offers a significant benefit to both the radial and azimuthal MTF for the same choice of Algorithm. For example, the 10% cutoff increases by approximately 35.86%, 32.58%, and 38.31% for the azimuthal MTF of the Bone, Bone Plus, and Edge algorithms when comparing normal and HR scan modes. It increases by approximately 19.75%, 19.48%, and 20.56% for the radial MTF. Though the HR scan mode does improve the azimuthal MTF more than the radial MTF, the azimuthal resolution remains worse than the radial resolution.

Figure 5 compares the Bone, Bone Plus, and Edge Algorithms with their HD counterparts while operating in the HR scan mode away from scanner isocenter. Figure 5 demonstrates that the Bone, Bone Plus, and Edge Algorithms performed similarly to their HD counterparts away from scanner isocenter. The mean 10% cutoffs for the azimuthal MTFs only show a statistically significant increase between Algorithms for Bone to HD Bone, with an increase of approximately 0.1 cycles/cm. While the difference in the means for the Edge and HD Edge algorithms in the azimuthal direction is approximately 0.5 cycles/cm, this difference is found to have a p‐value of 0.22 when performing a two‐tailed *t*‐test and is not statistically significant.

Meanwhile, the radial resolution does show some improvement due to Algorithm choice. The mean 10% cutoffs for the radial MTFs show statistically significant differences when a *t*‐test comparison is performed on the group means for the Bone and HD Bone Algorithms with an increase of 0.6 cycles/cm in MTF cutoff from Bone to HD Bone, and an increase in MTF cutoff of 1.75 cycles/cm from Edge to HD Edge Algorithms. The apparent discrepancy between the plots of Fig. 5 and the MTF cutoff value calculated in Table 3 is likely due to the fact that the HD Edge radial MTF away from isocenter exhibits a very gradual falloff in the tail and there is a large degree of uncertainty in the estimate of the spatial frequency of the MTF cutoff.

The HD Edge Algorithm exhibits a large amount of image noise, making MTF estimation less reliable without a great deal of averaging. While the HD Edge Algorithm significantly increases the image noise, all the HD Algorithms increase the noise in the image to some extent. Table 4 presents the noise measurements obtained in a uniform slice of the Catphan using a 40 mm^2^ circular ROI centered at the scanner isocenter. We see a large increase in image noise when using an HD Algorithm over its non‐HD counterpart, and only a slight increase in noise when changing from the normal mode to the HR scan mode.

**Figure 7:**
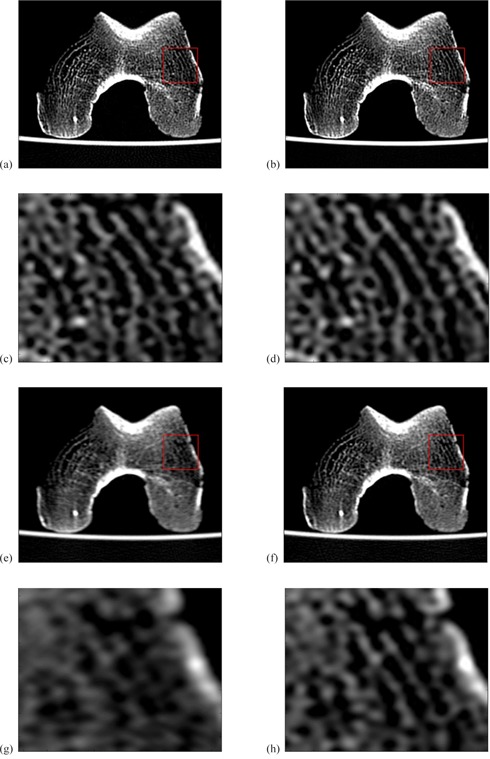
Images of a cadaveric human femur acquired at (a) and (b) scanner isocenter and (e) and (f) 15 mm away from image isocenter. WW/WL=2500/50. Figures (a) and (e) were acquired in the normal mode, and (b) and (f) were acquired in the HR scan mode. At isocenter the appearance of the images is comparable. Away from isocenter, the loss of fine detail in the image and the obvious blurring of the cancellous bone is easy to discern in the normal mode. Fine structures from (a) and (b) and (e) and (f) are highlighted in the zoomed in images of figures (c) and (d) and (g) and (h), respectively.

Figure 7 demonstrates the CT images of the cadaveric human femur. Figure 7(a), (c), (e), and (g) were acquired using the normal scan mode, while (b), (d), (f), and (h) were acquired in the HR scan mode. Images (a) to (d) were acquired with the femur positioned at scanner isocenter, while images (e) to (h) were acquired with the femur positioned 15 cm anterior to scanner isocenter.

**Table 4:** Noise measurements. The SD in a 40.0 cm^2^ ROI centered on scanner isocenter was measured across all images for a given scan mode and algorithm. Values are given in HUs.

	*Normal Mode*	*HR Mode*
Standard	1.5	1.6
Bone	5.6	6.5
Bone Plus	8.4	9.7
Edge	10.9	10.9
HD Bone		9.1
HD Bone Plus		15.9
HD Edge		38.8

## IV. DISCUSSION

It was found that the HR scan mode provided similar MTFs to the normal scan mode at scanner isocenter. However, the HD Algorithms provided a significant resolution increase compared with the non‐HD Algorithms at isocenter when using the HR scan mode. Away from isocenter, the HR scan mode offered a significant benefit to image resolution. However, utilizing higher resolution Algorithms, such as the HD Algorithms while in HR scan mode, provided little additional resolution improvement away from isocenter. Essentially, use of focal spot wobble affected image resolution much more than choice of Algorithm away from isocenter. Finally, regardless of scan mode or Algorithm, the degradation of MTF away from isocenter was worse for the azimuthal MTF than it was for the radial MTF.

The results just listed characterize the fall of the MTF toward the cutoff values given in Table 1. However, several of the algorithms provided by the scanner are ‘edge‐enhancing’ and rise above the value of 1 near the origin prior to falling off again. On the GE CT scanners, the Bone Plus Algorithm shows the greatest degree of edge enhancement. In Fig. 3(b), it was shown that, in addition to changing the cutoff values for the MTF, the HD Bone Plus Algorithm showed less Edge enhancement than the Bone Plus Algorithm. Additionally, the amount of edge enhancement for the Bone Plus Algorithm was reduced away from scanner isocenter, as can be seen in Fig. 4(b).

These results have practical, clinical consequences. The cadaveric human femur images show the loss of fine detail in the image and the blurring of the cancellous bone when in the normal scan mode away from isocenter. In the HR scan mode, there is still some loss in spatial resolution away from the scanner isocenter. However, fine details are much better preserved.

As demonstrated by the MTF curves shown in 3, 5, the HR scan mode and HD algorithms provide resolution improvements. However, use of the HD algorithms or the HR scan mode also results in a concomitant increase in image noise. The image noise in the HD Edge algorithm may be particularly prohibitive, with a measured noise of 38.8 HU compared to a measured noise of only 10.9 for the Edge algorithm in both normal and HR modes.

There are several applications that can benefit from increased image resolution, even at the expense of image noise, such as imaging the inner ear, imaging joints such as the knee or wrist, searching for nodules in the lungs, or examining stents in the coronary artery. The results in this manuscript demonstrate that every effort should be made to position extremities at scanner isocenter. The data in this paper are especially important to consider when repositioning a patient to achieve better centering is possible, but requires some motivation and effort.

While it has been shown that the optimal resolution is at scanner isocenter, use of the HR scan mode is found to increase spatial resolution throughout the image. This is important for situations when it is not possible to position the anatomy of interest at scanner isocenter. When trying to image a shoulder, a patient may have trouble lying on their side or contorting into an optimal position with respect to scanner isocenter. Additionally, some patients often have difficulty placing their elbow or wrist directly over their head, forcing a scan with their elbow or wrist positioned off‐centered.

The recommendation for clinical practice based on these findings is to always use HR mode when image sharpness is important away from the scan isocenter. To get optimal matching with the bowtie filter and accurate AEC control, patients should not be off‐centered in the CT scanner.^17^, ^18^, ^19^, ^20^ While this work does not support mispositioning patients in the CT scanner, it does provide a methodology to determine how near isocenter anatomy must be in order to achieve a particular resolution.

There are some limitations to this study. Resolution has only been measured at two points, and image noise has only been measured at isocenter when image resolution and image noise are changing continuously throughout the SFOV. However, measuring resolution over a large number of points throughout the entire SFOV would require too many measurements using the current technique, and a single‐noise measurement is sufficient to demonstrate relative noise levels of the different Algorithms and scan modes. Also, the noise analysis has not considered frequency dependence. The image resolution will also depend on the DFOV. The minimum possible DFOV of 5.0 cm was chosen in this study to limit the effect of voxel size on the MTF values. This study was concerned with resolution limitations due to the underlying hardware rather than blurring due to averaging within individual pixels. In some clinical scenarios, small DFOVs are used, for example in spinal or temporal bone anatomy imaging, making a 5.0 cm DFOV clinically relevant. Finally, image resolution may depend on the choice of axial versus helical mode and choice of slice thickness. A slice thickness of 5.0 mm was chosen in this study because utilizing a thinner slice would yield images with CT numbers that were above the maximum allowed by the system. In the most extreme cases, CT number saturation results in an artificial decrease in the MTF such that it falls off to zero more rapidly than it should if no saturation were present.^(21)^ Axial mode was chosen for this study so as to avoid any possibility of helical artifact.

## V. CONCLUSIONS

This study shows that, if resolution is critical for performing a diagnostic task, then on all scanners that have the capability a scan mode incorporating increased angular sampling and focal spot deflection should be used, regardless of the anatomy's position within the scanner relative to isocenter. This conclusion may be vendor‐independent, though this would need to be tested. Additionally, it was found that for the GE CT 750 HD, the use of the HR scan mode is more important than the choice of an HD versus regular Algorithm for image sharpness away from isocenter. To obtain very high image sharpness at isocenter and the best possible image sharpness over the image away from isocenter, these results show that HR scan mode with HD Algorithms should be used, while being careful in the choice of the Algorithm to avoid an unacceptable level of image noise.

## COPYRIGHT

This work is licensed under a Creative Commons Attribution 4.0 International License.
